# Challenges of Retained Thoracoamniotic Shunts in the Neonatal Period: A Case Report

**DOI:** 10.3390/children13020182

**Published:** 2026-01-28

**Authors:** Alejandro Madurga, María Victoria López Canelada, María Velayos, Carlos De la Torre, Eugenia Antolín Alvarado, Jose Luis Encinas, María Álvarez Barrial

**Affiliations:** 1 Department of Pediatric Surgery, La Paz University Hospital, 28046 Madrid, Spain; 2 Faculty of Medicine, Universidad Autónoma de Madrid, 28049 Madrid, Spain; 3 Department of Obstetrics and Gynecology, La Paz University Hospital, 28046 Madrid, Spain; 4 Department of Pediatric Surgery, King’s College Hospital NHS Foundation Trust, London SE5 9RS, UK

**Keywords:** thoracoamniotic shunting, fetal pleural effusion, retained shunt, shunt migration, neonatal thoracoscopy, recurrent pleural effusion, minimally invasive surgery

## Abstract

**Highlights:**

**What are the main findings?**

• Thoracoamniotic shunting can successfully resolve fetal hydrops and allow favorable perinatal outcomes, even in severe pleural effusions.
• Retained or migrated thoracoamniotic shunts may cause delayed postnatal complications, including recurrent pleural effusions, despite initial neonatal stability.

**What are the implications of the main findings?**

• Conservative observation is not always sufficient for retained thoracoamniotic shunts, and clinical deterioration should prompt active intervention.
• Thoracoscopic removal represents a safe, effective, and minimally invasive definitive treatment when postnatal complications arise.

**Abstract:**

**Background:** Thoracoamniotic shunting (TAS) is a well-established fetal therapy for severe pleural effusions complicated by hydrops. Although survival in selected cases exceeds 60%, retained or migrated shunts can pose significant postnatal management challenges. **Case presentation:** We report a neonate with intrathoracic migration of a Somatex^®^ shunt placed at 26 weeks’ gestation for hydropic pleural effusion. Although initially asymptomatic, the infant developed recurrent pleural effusions requiring multiple readmissions. Thoracoscopic retrieval on day 76 of life allowed safe removal despite dense adhesions, leading to complete clinical resolution. **Discussion:** Retained thoracoamniotic shunts may remain asymptomatic or cause recurrent effusions, pneumothorax, or other complications. This case highlights the limitations of conservative management in the presence of clinical deterioration and supports timely surgical removal. Standardized criteria for intervention are lacking and urgently needed. **Conclusions:** In infants with retained TAS, recurrence of effusions or respiratory compromise should prompt active removal. Thoracoscopic retrieval is a safe and effective minimally invasive option.

## 1. Introduction

Thoracoamniotic shunting (TAS) is an established fetal therapy for severe pleural effusions complicated by hydrops, aiming to decompress the thoracic cavity, promote lung development, and improve perinatal outcomes [1]. Reported survival rates in appropriately selected hydropic fetuses exceed 60%, making TAS a life-saving intervention [2]. Nevertheless, TAS carries inherent risks, including obstruction, occlusion, and migration, with intrathoracic displacement occurring in up to 20% of cases [3,4]. Postnatally, retained or migrated shunts present a management dilemma. While many series advocate conservative observation in asymptomatic infants, serious complications such as recurrent effusions, pneumothorax, or hilar strangulation have been reported [5,6]. Currently, there are no standardized guidelines for postnatal management, and practice varies considerably across centers. We present a neonate with intrathoracic migration of a Somatex^®^ shunt (Berlin, Germany), who developed recurrent pleural effusions necessitating thoracoscopic removal. This case highlights the clinical reasoning that justified active intervention and contributes to the ongoing debate regarding conservative versus surgical management of retained TAS.

## 2. Case Report

A 26-year-old primigravida with no relevant medical history was noted at 21 + 6 weeks’ gestation to have a fetus with bilateral hydrothorax, more pronounced on the left. Fetal thoracentesis and amniodrainage were performed, and fluid analysis revealed a cloudy, yellow exudate with 2510 nucleated cells/mm^3^, exhibiting a marked neutrophilic predominance (94%, 2359/mm^3^), scarce lymphocytes (1%), and occasional macrophages (5%). Despite laboratory findings, the leading suspected diagnosis was primary chylothorax. QF PCR and array analysis were negative for CMV and B19 Parvovirus, without finding pathogenic mutations (PTPN11 negative).

Serial ultrasounds demonstrated progression to hydrops at 26 + 2 weeks, prompting placement of a left-sided Somatex^®^ shunt. Subsequently, pleural effusions improved, and at 39 + 3 weeks a full-term vaginal delivery occurred.

Our Obstetrics department performed weekly ultrasounds, remarking on increasing polyhydramnios and diaphragmatic flattening, followed by a full hydrops picture at 26 + 2 weeks. Although there were no signs of heart failure, after discussing the case in our prenatal surgery committee, due to worsening fetal condition and parental wishes to continue the pregnancy, we chose thoraco-amniotic shunt placement as the best option. In this scenario and according to our previous experiences, we decided to insert a left Somatex^®^ shunt under ultrasound guidance.

**Figure 1:**
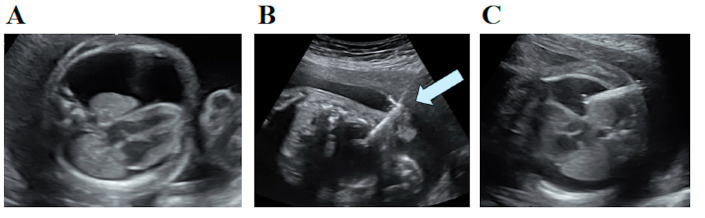
Prenatal ultrasound images (**A**) Bilateral hydrothorax larger on the left size detected at 21+6 weeks (**B**) Somatex^®^ shunt placement on left hydrothorax under ultrasound guidance (**C**) Control scan at 30+4 weeks.

Follow-up ultrasounds confirmed proper shunt placement and complete resolution of fetal hydrothorax. Weekly ultrasound evaluations after surgery were established to determine the proper placement and functioning of the shunt. We first had doubts about migration of the device just two weeks after the intervention at 29 + 6 weeks. However, at 30 + 4 weeks we found a stable right pleural effusion with minimal left effusion, dismissing shunt malfunction (Figure 1). At 37 + 4 weeks, pleural effusions were nearly resolved.

Finally, at 39 + 3 weeks, the baby was vaginally delivered, being a full-term newborn appropriate for gestational age. Weight: 3320 g (59th percentile), length: 51 cm (80th percentile), head circumference (HC): 35.8 cm (84th percentile).

**Figure 2:**
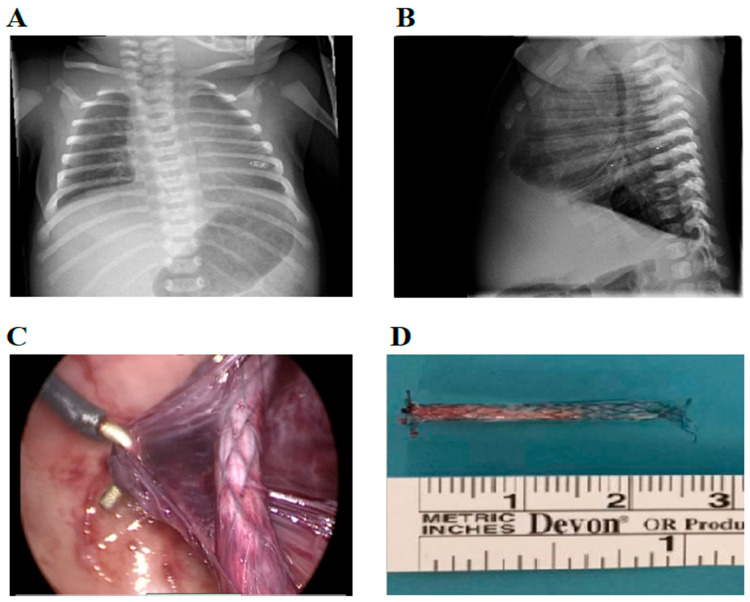
Postnatal chest radiography and surgical view of shunt removal via thoracoscopy. (**A**) Chest X-ray showing the shunt located inside de left hemithorax. (**B**) Lateral view showing costophrenic angle blunting revealing pleural effusion. (**C**) Thoracoscopic adhesiolysis and removal of device. (**D**) Somatex^®^ shunt outside the thoracic cavity showing its 25mm length, self-deploying parasols and nitinol wire mesh.

At birth, the shunt was not visible at the chest wall. Chest radiography confirmed intrathoracic migration (Figure 2A,B). The neonate was stable, with only mild transient respiratory distress. Echocardiography was normal. Following two days of observation in the neonatal intensive care unit (NICU), the infant was discharged for outpatient follow-up. During the first two months, recurrent pleural effusions required drainage and two NICU readmissions. After the final episode, surgical removal was scheduled and performed one week later, on day 76 of life.

The patient was placed in the right lateral decubitus position, and general anesthesia was induced. A monitor was placed over the patient’s head. We placed a 12 mm camera port in the 5th intercostal space aligned with the midaxillary line. Two additional 5 mm ports were inserted following a proper triangular arrangement. Carbon dioxide insufflation of 4 mmHg was achieved through one irrigation channel of the ports. Dense fibrous adhesions were encountered, preventing immediate identification of the thoracoamniotic shunt; therefore, meticulous blunt adhesiolysis was performed using thoracoscopic instruments, deliberately avoiding energy devices to reduce the risk of lung injury or air leak. After careful inspection of the thoracic cavity, we located the shunt outside the parenchyma, lying on the oblique fissure, and extracted it using an endoscopic grasping forceps inserted through the 5 mm port.

The removal of the device was conducted without any complications (Figure 2C,D). Follow-up radiographs demonstrated the complete absence of the shunt, accompanied by satisfactory pulmonary re-expansion. Chest radiographs were obtained every 24 h during the first three postoperative days, subsequently every three months during the first year, and then every six months until final discharge. The patient was discharged after a four-day hospitalization. Following discharge, the patient was monitored over a two-year period, during which regular control radiographs were performed. Lung ultrasound may be used for follow-up; however, it requires experienced pediatric radiologists and remains operator dependent, which may limit its reliability. Throughout this follow-up period, there were no recurrent pleural effusions observed, nor was there any indication of the development of pulmonary pathologies. Consequently, it was determined that the patient could be discharged from our outpatient clinic.

## 3. Discussion

Thoracoamniotic shunting has become an established prenatal intervention for large fetal pleural effusions, particularly when associated with hydrops, as it aims to restore cardiopulmonary physiology and prevent progressive pulmonary hypoplasia. Despite its widespread use, the postnatal management of retained thoracoamniotic shunts remains poorly standardized, largely due to the rarity of reported complications and the absence of prospective studies. Most available evidence derives from retrospective series and isolated case reports, resulting in heterogeneous recommendations regarding observation versus elective removal.

Fetal pleural effusions can be managed prenatally by either thoracentesis (drainage) or placement of a pleuro-amniotic shunt. In our center, the presence of hydrops is an indication for pleuro-amniotic shunt insertion following drainage of the effusion. Leaving a shunt in situ allows continuous drainage of pleural fluid into the amniotic cavity, thereby reducing the risk of prenatal recurrence and the need for repeated invasive procedures.

Early neonatal outcomes after thoracoamniotic shunting can vary significantly, and assessing the initial postnatal findings is crucial for guiding further management. Variations in respiratory transition, the persistence or resolution of effusions, and the position of the shunt at birth all offer important insight into the effectiveness of the fetal intervention and the potential risk of subsequent complications.

The shunt was placed on the left side, as seen on the ultrasound images, where the pleural effusion was largest and there was sufficient space for insertion. Prenatally, there is no fixed anterior or lateral location for shunt placement, as the procedure is ultrasound-guided and the shunt is inserted at the site with the maximum fluid collection. The available space is often limited, and fetal position is usually the main constraint. The Somatex shunt was chosen because it is the only shunt currently available at our center. Although other shunt types can be used, Somatex is preferred at our center, and in our experience the rate of dislodgement is low. The shunt was placed at 26 + 2 weeks’ gestation, at a time when hydrops was present, including pleural effusions and generalized skin edema.

Insertion of a Somatex^®^ thoracoamniotic shunt led to resolution of fetal hydrops and a favorable pregnancy course, resulting in term vaginal delivery. This outcome supports the efficacy of thoracoamniotic shunting (TAS) in selected cases, as previously reported with survival rates above 60% in hydropic fetuses [1,2].

Although TAS offers significant benefits, it may also cause obstruction, occlusion, or migration, with intrathoracic migration occurring in up to 20% of cases [3,4]. Even with the Somatex^®^ shunt—designed to reduce displacement—migration can still occur [5,6]. In our case, postnatal imaging confirmed migration into the left hemithorax. The newborn was initially asymptomatic, justifying conservative management [3,4]. However, two episodes of recurrent pleural effusion within the first two months indicated clinical deterioration, prompting surgical removal. Similar reports describe retained shunts causing persistent effusions, pneumothorax, or even hilar strangulation [7,8,9].

Postnatal management strategies for retained intrathoracic shunts range from expectant observation in asymptomatic neonates to early surgical retrieval. Conservative management has traditionally been favored in clinically stable infants, based on earlier reports suggesting minimal long-term risk. However, this approach relies heavily on the assumption that retained devices remain inert. Increasing evidence challenges this assumption, as delayed complications may develop after an initial period of apparent stability, making exclusive reliance on early postnatal findings potentially misleading.

Thoracoscopic removal was successfully performed despite dense adhesions. This approach is supported by increasing evidence showing that minimally invasive retrieval is both safe and effective, offering low morbidity and rapid recovery [7,10,11]. Consistent with these findings, the patient remains asymptomatic with normal pulmonary development at two-year follow-up.

This case contributes to the ongoing discourse regarding the optimal management of retained thoracoamniotic shunts (TAS). While earlier retrospective studies suggest that shunts can remain in place without long-term harm [3], more recent evidence highlights the risk of delayed complications, including recurrent effusions, pneumothorax, and life-threatening events associated with long-term device retention. Two perspectives are evident in the current literature. On one hand, large retrospective series suggest that retained intrathoracic shunts may remain in situ without adverse outcomes, with follow-up extending into early adulthood in some cases [3]. On the other hand, more recent reports highlight the potential for delayed and unpredictable complications—including recurrent effusions, pneumothorax, and even life-threatening events—associated with long-term device retention [7,8,9]. Our case supports the latter approach. Following the onset of recurrent pleural effusions, conservative management was no longer appropriate in our case, and surgical removal led to complete clinical resolution. These findings emphasize the importance of timely surgical intervention in appropriately selected cases.

Our case highlights that the postnatal course of infants with retained thoracoamniotic shunts can change rapidly and that relying solely on initial stability may delay necessary treatment. Systematic follow-up with the use of predefined clinical and radiological checkpoints would help detect early signs of deterioration and guide timely referral for removal. Developing standardized criteria for intervention based on accumulated clinical experience and reported complications would reduce variability between centers and support earlier, evidence-based decision-making.

From a clinical perspective, the development of standardized postnatal surveillance protocols incorporating predefined clinical and radiological criteria could reduce variability between centers and facilitate earlier, evidence-based decision-making. Future research should focus on multicenter registries and long-term outcome studies to better define risk factors for delayed complications, clarify the optimal timing for elective removal, and establish consensus guidelines for postnatal management of retained thoracoamniotic shunts.

## 4. Conclusions

Retained or migrated thoracoamniotic shunts should not be considered inert remnants of prenatal therapy but rather dynamic foreign bodies with the potential to drive ongoing postnatal pathology. While thoracoamniotic shunting remains an effective fetal intervention, the clinical challenge shifts postnatally when the device is not removed, transforming a life-saving prenatal tool into a possible source of pleural inflammation, adhesion formation, and recurrent effusions.

An important and potentially misleading aspect of postnatal management is the reliance on early clinical stability as a marker of resolution. This case illustrates a clear temporal dissociation between initial postnatal well-being and the later emergence of shunt-related complications. An apparently uneventful neonatal course may therefore mask a progressive pathological process, creating a false sense of security and contributing to delayed decision-making. Recurrence of pleural effusion in this context should be interpreted as a sentinel event, signaling persistent interaction between the retained shunt and the pleural environment rather than as an isolated or self-limited finding.

Thoracoscopic removal represents more than a minimally invasive technical solution; it constitutes a definitive intervention that addresses the underlying mechanism of disease. Even in the presence of fibrous adhesions, thoracoscopy allows direct visualization of the pleural cavity, facilitates safe extraction, and enables both diagnostic assessment and therapeutic resolution. Importantly, earlier intervention may reduce the progression of pleural damage and avoid the increased technical complexity associated with delayed surgery.

At present, postnatal management strategies for retained thoracoamniotic shunts remain highly variable and largely dependent on institutional experience and individual clinician judgment. This variability reflects the absence of standardized postnatal algorithms rather than true differences in disease behavior. Structured follow-up protocols incorporating clinical evaluation, serial imaging, shunt position or migration, and the recurrence of pleural effusions could help identify patients who would benefit from early and proactive surgical removal. In practical terms, a low threshold for intervention should be maintained when recurrent effusions, migration, or inflammatory sequelae are identified, rather than prolonged watchful waiting.

Ultimately, this case underscores the need to reframe postnatal care of thoracoamniotic shunts as an active surveillance and decision-making process rather than a passive observational strategy. Multicenter collaboration and dedicated registries will be essential to refine risk stratification, inform evidence-based timing of intervention, and improve outcomes in this rare but clinically significant condition.

## Data Availability

The original contributions presented in this study are included in the article. Further inquiries can be directed to the corresponding author.

## Abbreviations

The following abbreviations are used in this manuscript:

TAS	Thoracoamniotic Shunting
NICU	Neonatal Intensive Care Unit
